# Alcohol-Induced Mesenteric Lymphatic Permeability: Link to Immunometabolic Modulation of Perilymphatic Adipose Tissue

**DOI:** 10.3390/ijms20174097

**Published:** 2019-08-22

**Authors:** Flavia M. Souza-Smith, Liz Simon, Robert Siggins, Patricia E. Molina

**Affiliations:** Department of Physiology, LSUHSC-New Orleans, New Orleans, LA 70112, USA

**Keywords:** lymphatic leakage, ethanol, mesenteric fat, immunometabolism

## Abstract

Alcohol exerts significant immunomodulatory effects on innate and adaptive immune responses, impairing host defense against infections. Gut-mucosa-derived dendritic cells (DCs) traffic to mesenteric lymph nodes (MLNs) through mesenteric lymphatic vessels (MLVs), contributing to intestinal antigen homeostasis. Previously, we demonstrated that acute alcohol administration to male rats induces MLV hyperpermeability resulting in perilymphatic adipose tissue (PLAT) inflammation and insulin signaling dysregulation. We hypothesized that alcohol-induced MLV hyperpermeability can lead to DC leakage to PLAT. DCs promote adipose tissue regulatory T cell (Treg) expansion, and this has been proposed as a mechanism underlying age-associated insulin resistance (IR). The aim of this study was to determine whether chronic alcohol consumption promotes DC leakage to PLAT and results in metabolic dysregulation. Male rats received a Lieber–DeCarli liquid diet containing 36% of calories from alcohol for 10 weeks. Time-matched control animals were pair-fed. PLAT, MLNs, and peripheral blood leukocytes (PBLs) were isolated for flow cytometry analyses. PLAT explants were used for determinations of insulin-induced glucose uptake. Chronic alcohol consumption decreased MLN CD4/CD8 ratio and Treg frequency in PBLs. Alcohol increased the frequency of DCs, CD4 T cells, and Tregs in PLAT. Lastly, alcohol decreased insulin-stimulated glucose uptake in PLAT. Collectively, these findings suggest that alcohol-induced immune cell deviation from the gut–MLN pathway is associated with PLAT immunometabolic dysregulation. Whether this immune cell deviation impacts induction of mucosal immunity warrants further investigation.

## 1. Introduction

Alcohol consumption is prevalent in the US, with over 50% of adults over 18 years old being regular drinkers [1]. Among the several consequences of acute and chronic alcohol consumption, the immunomodulatory effects of alcohol affecting innate and adaptive immune responses contribute to impaired antimicrobial defense and inflammatory responses throughout the body. As a consequence, alcohol impairs host response to numerous infections or worsens the effects of disease affecting several organs and tissues [2]. The immune system plays a comprehensive and understated role in adipose tissue and systemic metabolism surveilling and responding to specific metabolic signals through a set of processes termed immunometabolism [3]. Chronic and binge alcohol drinking have been shown to impact the whole body and adipose tissue by impairing insulin-dependent responses and decreasing insulin-stimulated glucose uptake [2,4,5]. Although reports of alcohol’s immunomodulatory effects are abundant in the literature, an immunometabolic link remains to be fully examined. Consequently, the mechanisms underlying alcohol-induced immune cell-mediated metabolic homeostasis in the adipose tissue are unknown.

The lymphatic vessels are important conduits for antigen transport and integrity of immune cell regulatory functions and can undergo changes in permeability during insult [6]. At steady state, antigen-presenting cells, such as dendritic cells (DCs), leave the gut via mesenteric lymphatic vessels (MLVs) and travel into mesenteric lymph nodes (MLNs) to participate in host immune responses [7]. The mesenteric perilymphatic adipose tissue (PLAT), the rodent visceral adipose tissue surrounding all collecting lymphatic vessels, is the primary target for leaked macromolecules from lymphatic vessels. Infection-induced mesenteric lymphatic leakage leads to DC deviation out of collecting lymphatic vessels and into PLAT, interrupting the immune dialogue in between the gut and MLNs [8]. DCs not only bring antigens into PLAT but also stimulate T cell expansion into fat-resident regulatory T cell (Treg) [9]. The presence of a distinct population of Tregs is found in several non-lymphoid tissues including adipose tissue; they are distinct from the classical lymphoid organ in phenotype and function [10]. The Tregs are crucial for maintaining self-tolerance but their functional mechanisms in non-lymphoid tissues are poorly understood. At present, one of the best characterized non-lymphoid tissue-resident Tregs is the population found in the visceral adipose tissue [11]. Interestingly, a large percentage of visceral adipose tissue CD4+ cells are Tregs expressing forkhead box P3 (FOXP3), a much larger fraction than that found in lymphoid or non-lymphoid tissues such a subcutaneous fat (SFAT) [10]. Remarkably, an increase in visceral adipose tissue Tregs has been proposed as the adipo-immune driver of age-associated insulin resistance, as Treg depletion in adipose tissue increased insulin sensitivity [12]. Additionally, fat-Treg-deficient mice were shown to be protected against age-associated insulin resistance [12]. 

We have demonstrated that acute alcohol administration to rats leads to mesenteric collecting lymphatic leakage, PLAT immune cell recruitment and inflammation, and impairments in insulin signaling specifically in PLAT and not in other adipose depots, including SFAT [13,14]. Thus, not only is PLAT a potentially critical tissue involved in alcohol-mediated immunomodulation but it may also be vulnerable to alcohol-induced insulin resistance similar to that characterizing insulin resistance of aging. To expand our understanding of the interaction of immune macromolecules contained in MLVs with PLAT, this study aimed to investigate the effects of a chronic alcohol diet on PLAT immunometabolism in rodents.

## 2. Results

### 2.1. Chronic Alcohol Increases Lymphatic Permeability

**Figure 1:**
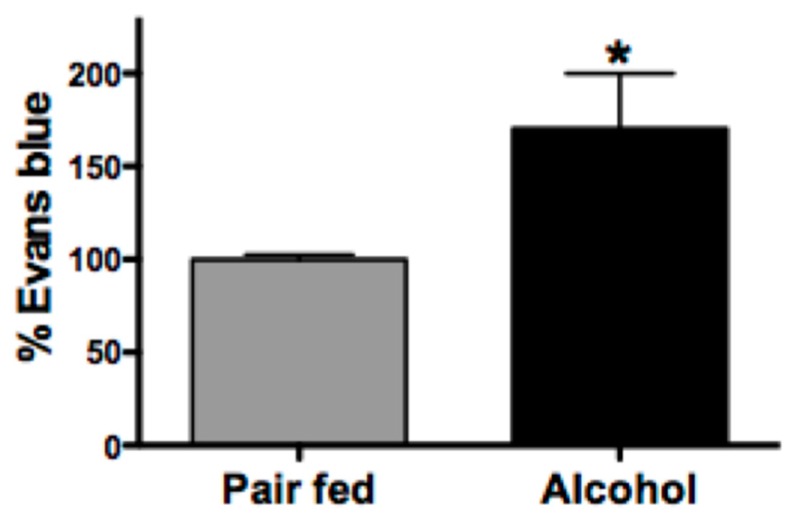
Percent (%) of Evans blue leakage into perilymphatic adipose tissue (PLAT). Alcohol increases the permeability of lymphatic vessels to Evans blue in mesenteric adipose tissue (MFAT) compared with pair-fed animals. T-test (*n* = 4–5), T-test, Mean ± SEM, * *p* < 0.05.

We have previously demonstrated that a single bolus or repeated binge alcohol administration leads to mesenteric collecting lymphatic hyperpermeability and leakage into PLAT as determined by Evans blue extravasation into PLAT [13,14]. After 10 weeks of alcohol or control diet, there was a significant increase in leakage of intragastrically administered Evans blue out of mesenteric collecting lymphatic vessels and into PLAT (Figure 1, *n* = 4–5, *p* < 0.05). Consistent with our previous reports, these data suggest alcohol-induced lymphatic leakage.

### 2.2. Chronic Alcohol Decreases the CD4+/CD8+ T Cell Ratio

**Figure 2:**
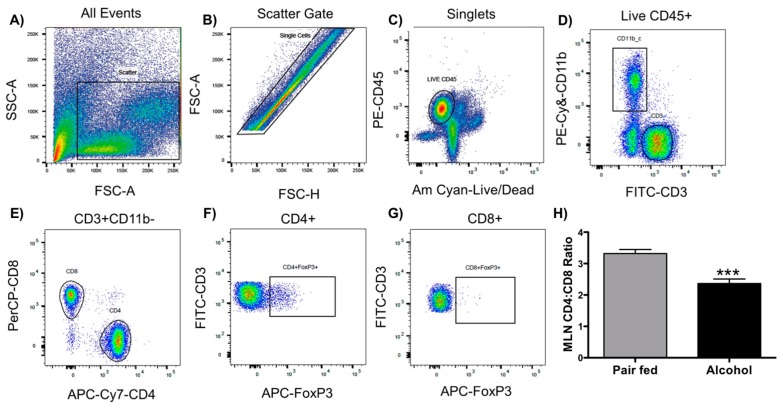
Flow-cytometry-gating strategy (**A**–**G**). Ratio of CD4 to CD8 T cells in MLNs (**H**). Alcohol decreases the ratio of CD4/CD8 T cells in the mesenteric lymph nodes (MLNs) compared with pair fed animals (H). T-test (*n* = 6), T-test, Mean ± SEM, *** *p* < 0.001.

**Figure 3:**
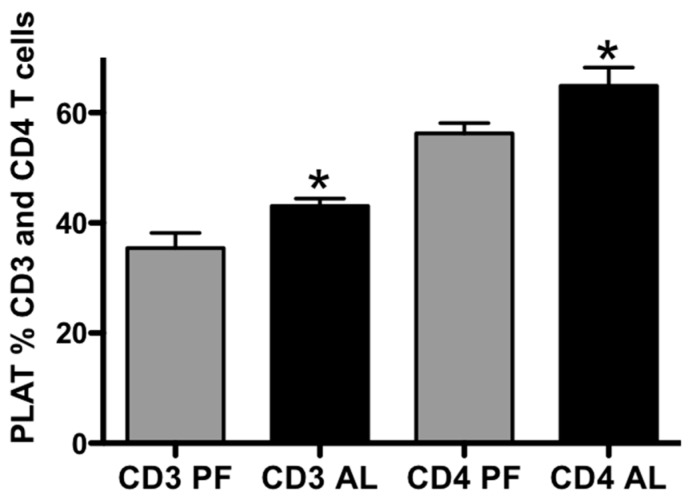
Percent (%) of CD3 and CD4 T cells in PLAT. Alcohol (AL) increased % CD3 and CD4 T cells in the MFAT compared with pair-fed (PF) animals. T-test (*n* = 6), T-test, Mean ± SEM, * *p* < 0.05. These data were generated using the gating strategy shown in Figure 2.

**Figure 4:**
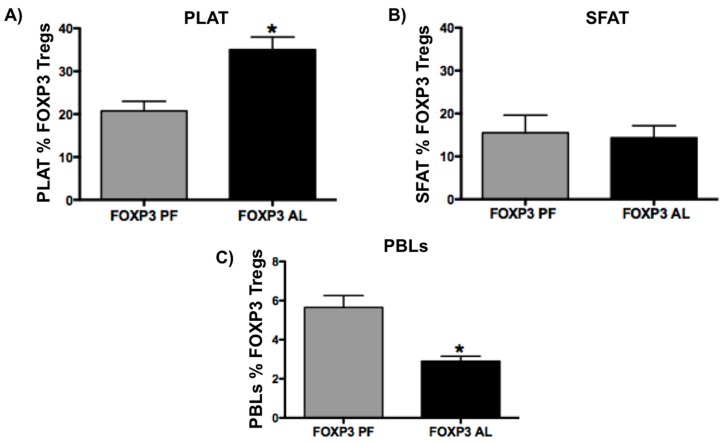
Percent (%) of Tregs in PLAT (**A**), subcutaneous fat (SFAT) (**B**), and peripheral blood lymphocytes (PBLs) (**C**). Alcohol (AL) increased % Tregs (FOXP3) in PLAT (**A**), did not alter it in SFAT (**B**), and decreased % Tregs in PBLs (**C**) compared with pair-fed (PF) animals. T-test (*n* = 6), T-test, Mean ± SEM, * *p* < 0.05. These data were generated using gating strategy shown on Figure 2.

Figure 2A–G presents a flow-cytometry-gating strategy to generate data for Figure 2, Figure 3 and Figure 4. To assess T cells in the MLNs, lymphocytes were isolated from MLNs from alcohol- and pair-fed animals. Our results show that alcohol significantly decreased the ratio of CD4+ T cells to CD8+ T cells compared with pair-fed animals (Figure 2, *n* = 6, *p* < 0.001). A decreased CD4/CD8 ratio has been shown by others to be associated with decreased host resistance to infection [15]. 

### 2.3. Chronic Alcohol Increases T Cells and Tregs in PLAT and Decreases Percent Tregs in Peripheral Blood Leukocytes (PBLs)

Vascular endothelial cells, macrophages, and lymphocytes are found in the stromal vascular fraction. Flow cytometry was used to determine the phenotype of lymphocytes (surface markers for CD3+ and CD4+ T cells and transcription factor FOXP3 (Tregs)) isolated from PLAT and SFAT stromal vascular fraction. Alcohol significantly increased total CD4+ T cells (Figure 3, *n* = 6, *p* < 0.05) and Tregs (Figure 4A, *n* = 6, *p* < 0.05) in PLAT stromal vascular fraction, while it did not change total CD4+ T cells (data not shown) or Tregs in SFAT (Figure 4B, *n* = 6) when compared with pair-fed controls. Interestingly, chronic alcohol did not change the total CD4+ T cells (data not shown) but significantly decreased the percent of Tregs in PBLs (Figure 4C, *n* = 6, *p* < 0.05) when compared to pair-fed controls. These data suggest that the increased Tregs in PLAT may not be derived from the circulation but may result from expansion in PLAT.

### 2.4. Chronic Alcohol Increases Dendritic Cells (DCs) in PLAT and Decreases DCs in MLNs

**Figure 5:**
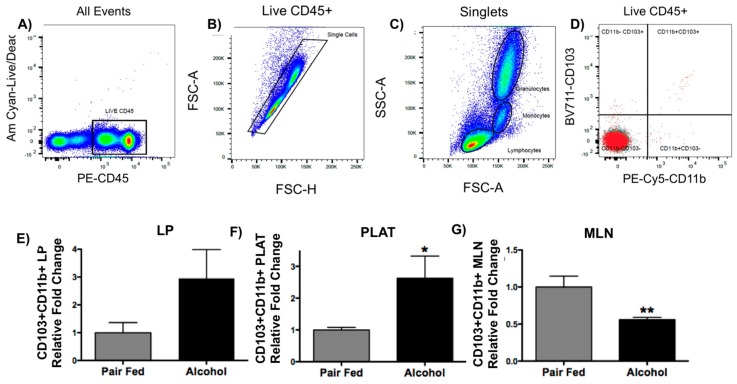
Flow-cytometry-gating strategy (**A**–**D**). Relative change in DCs in intestinal lamina propria (LP) (**E**), PLAT (**F**), and MLNs (**G**). Alcohol produced a relative increase in DCs in LP (**E**) and PLAT (**F**) and a relative decrease in MLNs (**G**) compared with pair-fed animals. T-test (*n* = 4 LP; *n* = 6 PLAT; *n* = 7–8 MLNs), T-test, Mean ± SEM, * *p* < 0.05, ** *p* < 0.01.

DCs were measured in the intestinal lamina propria (LP), in the stromal vascular fraction of PLAT, and in the MLNs. CD103+CD11b+ DCs are migratory and have been associated with induction of Treg expansion [16]. The flow-cytometry-gating strategy is shown on Figure 5A–D. Alcohol produced a modest increase in LP DCs, but failed to reach statistical significance (Figure 5A, *n* = 4) when compared with controls. Alcohol significantly increased DCs in PLAT (Figure 5B, *n* = 6, *p* < 0.05) and decreased DCs in MLNs (Figure 5C, *n* = 7–8, *p* < 0.01) when compared with pair-fed controls. These data suggest that the deviation of DCs into PLAT, via lymphatic leakage, could be a mechanism of PLAT Treg expansion. Moreover, we speculate that MLV leakage of DCs into PLAT precludes them from reaching the MLNs and may interfere with induction of proper immune responses.

### 2.5. Chronic Alcohol Decreases Glucose Uptake in PLAT 

**Figure 6:**
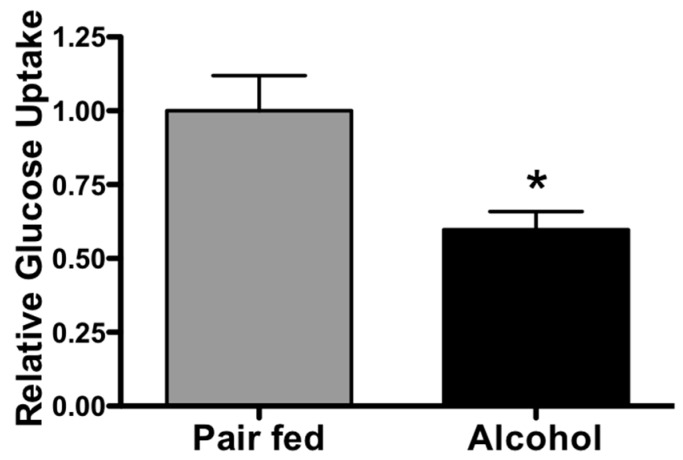
Relative glucose uptake. Alcohol (AL) decreases glucose uptake in PLAT compared with pair-fed (PF) animals. T-test (*n* = 7–8), T-test, Mean ± SEM, * *p* < 0.05.

To determine whether alcohol-induced lymphatic leak and increased PLAT Tregs were associated with evidence of metabolic dysregulation, we examined insulin-mediated responses in adipose tissue (PLAT and SFAT explants). Our results show that chronic alcohol significantly decreased glucose uptake in PLAT (Figure 6, *n* = 7–8, *p* < 0.05) but not in SFAT (pair-fed 1 ± 0.159 and alcohol 1.159 ± 0.344, *p* = 0.69). 

## 3. Discussion

We examined the effects of chronic alcohol consumption on adipose tissue immunometabolism. The data showed that chronic alcohol leads to increased MLV permeability, allowing DCs to leak into the PLAT, interrupting the traffic of immune cells between the gut and the MLNs. These results suggest this may be the underlying mechanism of the decreased CD4/CD8 ratio in the MLNs seen in alcohol-fed rats. Our results showed decreased glucose uptake in PLAT of alcohol-fed rats, suggesting alcohol-induced immunometabolic dysregulation. We speculate that these early alterations in metabolic regulation may contribute to the eventual onset of systemic alcohol-induced insulin resistance. Furthermore, we hypothesize that alcohol-induced deviation of DCs into the PLAT may chronically disable the induction of mucosal immunity and lead to defective host response to infection. 

Our previous studies show that a single alcohol bolus or repeated binge-like alcohol intoxication promotes mesenteric lymphatic hyperpermeability and lymphatic leakage into PLAT, increased PLAT macrophages, DCs, and markers of inflammatory cytokines. In addition, we found that alcohol promotes innate immune cell recruitment to PLAT, decreases circulating adiponectin levels, and impairs PLAT insulin signaling; implying a potential role of alcohol on adipose immunometabolic dysregulation [13,14]. Collectively, these findings support an immune crosstalk between lymphatic vessels and adipose tissue. The profound modulatory effects of alcohol on the immune system observed in our previous studies were acute. The effects of chronic alcohol consumption on PLAT immunometabolism had been previously unexplored. The results from these studies confirmed that chronic alcohol consumption significantly increases lymphatic permeability, alerting to the possibility that alcohol consumption results in chronic leakage of gut-derived macromolecules into PLAT. One of the critical functions of the mesenteric lymphatic system is to promote immune surveillance by transporting immune cells and antigens from the gut to the lymph nodes [7]. Remarkably, recent findings demonstrated that collecting lymphatic vessels permeability facilitates adipose tissue inflammation by allowing broad distribution of lymph components within the surrounding fat [17]. Therefore, we anticipate that chronic alcohol-induced lymphatic leakage leads to immune cell and antigen outflow into the PLAT, sequestering these cells from reaching the MLNs, preventing the onset of an immune response.

The CD4/CD8 ratio is a hallmark of immune cell disturbance and immune suppression. This ratio reflects T cell derangements associated with aging and can be a predictor of mortality in the general population. It is also widely used in human immunodeficiency viruses (HIV) patients as a morbidity and mortality predictor [15]. Alcohol use can modify the function, amount, and survival of most immune cells, and long-term use of alcohol increases the risk and severity of infections [18]. A canonical immune response involves the activation of T cells by DCs allowing these T cells to proliferate, differentiate, and migrate to infection sites. Our findings showed that 10 weeks of alcohol consumption significantly decreased the CD4/CD8 ratio in the MLNs. Although this MLN decreased ratio might not be sufficient to adversely affect health, we predict that it might decrease the body’s resistance to infection. Furthermore, it is possible that alcohol-induced lymphatic leakage allows leakage of immune cells into the PLAT, specifically gut-derived antigen-presenting DCs travelling to the MLNs. This could prevent MLN CD4 T cell activation and proliferation, contributing to the observed decreased CD4/CD8 ratio. 

Although adipose tissue Tregs have been shown to play an important role in metabolic dysregulation, data regarding the abundance of Tregs in the visceral adipose tissue are conflicting. For example, decreased numbers of Tregs are found in visceral adipose tissue of obese mice, and this decrease is associated with insulin resistance [10]. In obese patients with insulin resistance, the amount of Tregs in visceral adipose tissue is increased in comparison to lean controls [19]. Finally, recent findings demonstrated that adipose-tissue-resident Tregs accumulate in visceral depots as a function of age, but not obesity, and selective depletion of adipose-resident Tregs in these aged animals enhanced adipose tissue insulin sensitivity [12]. Here we found that after 10 weeks of alcohol diet consumption, PLAT Tregs significantly increased compared with pair-fed controls, while the percent of circulating Tregs decreased. Although we cannot rule out that PLAT Tregs can be derived from the circulation, we believe that Tregs are being locally expanded for two reasons. First, there are no differences between alcohol and pair-fed Tregs in SFAT and second is that the percentage decrease of Tregs in the PBLs after 10 weeks of alcohol, although significant, is about 2.7% while the percentage increase of fat Tregs in PLAT at the same conditions is 14.4%. Transcriptionally, fat Tregs were shown to be more closely clustered with fat CD4+ T cells than splenic Tregs, suggesting that the functional specification of fat Tregs is informed by their anatomical location within adipose tissue [12]. Additionally, studies suggest that visceral adipose tissue Tregs are a unique population of fat-resident cells with a distinct transcriptome and antigen receptor repertoire in comparison to their lymphoid-tissue counterparts. Furthermore, in agreement with our findings, fat Tregs have a higher fractional representation being a higher percentage of CD4 + T cells in visceral fat than in the blood [10]. Finally, classic in vitro suppression assays have shown the ability of Tregs to abrogate lymphocyte proliferation [20]. The fact that we also found increased CD3 and CD4 T cells in PLAT from chronic alcohol-treated animals suggest that the increased fat Tregs may not be suppressing fat CD4 T cell proliferation. The consequences of alcohol-induced PLAT Tregs are still to be determined. We predict these may involve similar mechanisms to those that characterize age-induced fat Treg expansion and consequently age-associated insulin resistance.

The main route for DCs to enter lymph nodes is via collecting lymphatic vessels, which carry the DC and its precursors in addition to transporting antigens [7]. Recent findings showed that sustained inflammation and associated lymphatic leakage into PLAT deviates migratory gut-derived DCs, circulating through lymphatic vessels, to the PLAT, preventing their accumulation in the MLNs. Consequently, we predict that the conventional immune functions (tolerance and protective immunity) are persistently compromised [8]. DCs are known to be strong stimulators of Treg expansion. Gut immunity is mediated by antigen presenting cells that can be grouped based on their expression of integrin αE (CD103) and αM (CD11b) [8]. The intestinal DC subset with the most potent Treg-inductive capacity expresses CD103+CD11b+ and is a migratory DC subset [16]. Here, these subsets were measured in the intestinal lamina propria, PLAT, and MLNs. Alcohol increased the migratory DCs in the intestinal lamina propria and PLAT, but significantly decreased DCs in the MLNs. These data suggest that alcohol-induced lymphatic leakage is permissive for intestinal DC leakage into PLAT and, potentially, for local stimulation of Treg expansion. Moreover, leakage of DCs into PLAT prevents enough DCs from entering the MLNs, which we believe may impair appropriate immune responses. Thus, these findings provide insight into the role of chronic alcohol on DC trafficking and migration, and future studies will determine whether DCs directly stimulate Treg expansion.

A major metabolic defect associated with insulin resistance is the failure of peripheral insulin sensitive tissues, such as, adipose, muscle, and liver to properly utilize glucose. Chronic alcohol decreases insulin-stimulated glucose uptake in adipose and muscle [21]. In addition, our previous findings showed that a three-day repeated binge-like alcohol intoxication impaired insulin signaling in PLAT but not in SFAT, suggesting depot-specific alterations in adipose tissue homeostasis [13]. Here, we determined whether alcohol-induced lymphatic leakage and increased PLAT Tregs were associated with signs of metabolic dysregulation, by measuring insulin-mediated responses in PLAT explants. While there were no effects of alcohol on glucose uptake in SFAT (data not shown), we found that chronic alcohol significantly decreased glucose uptake in PLAT. The early occurrence of insulin resistance in the adipose tissue is thought to precede whole-body insulin resistance [22]. Interestingly, although weight loss can reverse several parameters, such as, hypertension, dyslipidemia, hyperglycemia, systemic inflammation, and insulin resistance in the whole-body, muscle, and liver, weight loss cannot reverse adipose tissue insulin resistance [22]. Together with our previous findings, we predict that the early alterations in metabolic regulation shown here contribute to the eventual onset of systemic alcohol-induced insulin resistance. 

In summary, chronic alcohol leads to mesenteric lymphatic leakage, DC leakage into PLAT, Treg expansion in PLAT, decreased CD4/CD8 ratio in the MLNs, and decreased glucose uptake by PLAT. Overall, the findings suggest an alcohol-induced immunometabolic dysregulation. These findings advance our understanding, but also raise several questions that warrant further investigation. For example, the mechanisms underlying alcohol-induced lymphatic vessel leakage are unidentified and will be investigated. Moreover, the mechanisms of increased Tregs in PLAT, as well as the potential role of PLAT Tregs contributing to the risk for development of insulin resistance, remain to be elucidated. 

## 4. Materials and Methods 

### 4.1. Animals and Diet

Animal studies were approved by the Institutional Animal Care and Use Committee at the Louisiana State University Health Sciences Center (#3400, 10/12/2019, Approved by LSUHSC’s Institutional Animal Care and Use Committee) and were performed in accordance with the guidelines of the NIH *Guide for the Care and Use of Laboratory Animals* (8th edition, 2011). Male Fisher 344 rats (180–200 g body weight) were housed in a controlled temperature (22 °C) and controlled illumination (12:12 h light–dark cycle) environment. After arrival, the rats were allowed a one-week acclimation period and were provided standard rat chow (2018 Teklad Global 18% Protein Rodent Diet, Harlan, Riverside, CA, USA) and water ad libitum for the first week. The animals were transitioned into the Lieber–DeCarli liquid diet (BioServ, Flemington, NJ, USA) and solid food was decreased over five days. The animals were randomized to either alcohol-fed or pair-fed control group. Rats on the alcohol diet initially received 12% of total calories from alcohol and this percentage was increased every 4–5 days to 24% and finally 36% of caloric intake from alcohol. Time-matched pair-fed control rats received a liquid diet where maltose-dextran was isocalorically substituted for alcohol. The animals were kept on 36% alcohol diet for 10 weeks and were sacrificed for tissue collection. Animal body weights in the end of the study were not different between the groups (pair-fed 249 ± 5.359 g and alcohol 247.9 ± 5.647 g). This protocol of chronic alcohol intake achieves blood alcohol levels of ~100–150 mg/dL, and the animals continue to gain weight and grow.

### 4.2. Cell Isolation

After 10 weeks, animals were anesthetized with ketamine/xylazine, 90/9 mg/kg and blood, mesenteric fat (PLAT), subcutaneous fat (SCAT), mesenteric lymph nodes (MLNs), and small intestine were collected for cell isolation. 

Blood was mixed with 1 mL RBC Lysis Buffer (Qiagen, Germantown, MD, USA) for 10 min, centrifuged (500*× g*/5 min, room temperature (RT)) and the cells were washed in 1X phosphate-buffered saline (PBS) two times and re-suspended in 500 µL of PBS for flow cytometry.

Mesenteric and subcutaneous fat stromal vasculature fractions were isolated. The fat was washed in PBS four times, minced, and incubated in 15 mL of PBS and 20 mg of collagenase Type I (Sigma, St. Louis, MO, USA) at 37 °C, 5% CO_2_ in a shaker for 30 min. PBS (10 mL) was added and the mix was pipetted several times to break up aggregates. The liquid portion was transferred into a 50 mL tube leaving the solids behind by passing through a cell strainer (70 mm). The cells were washed twice in PBS and re-suspended in 1 mL of RBC Lysis Buffer and incubated at RT for 10 min. Five milliliters of PBS was added and the cells were centrifuged (500*× g*/5 min, RT) and washed in PBS twice. Cells were re-suspended in 500 µL of PBS for flow cytometry.

Mesenteric lymph nodes were smashed/strained through a cell strainer (70 mm) with a plunger of a syringe and washed with 10 mL of PBS. The cells were washed twice in PBS and re-suspended in 500 µL of PBS for flow cytometry.

The intestinal tract was cut longitudinally, cleaned in PBS and segmented into 1 cm fragments. Fragments were placed in 20 mL of Hank’s balanced salt solution (HBSS) at 37 °C, 5% CO_2_ in a shaker for 30 min. Fragments were transferred into 20 mL HBSS and 15 mg of collagenase Type I and placed at 37 °C, 5% CO_2_ in a shaker for 30 min. The intestinal segments were smashed/strained through a cell strainer (70 mm) with a plunger of a syringe. The cells were washed four times with 10 mL of PBS and re-suspended in 500 µL of PBS for flow cytometry.

### 4.3. Lymphatic Permeability

We used a separate cohort of animals fed a Lieber–DeCarli diet for 10 weeks to assess lymphatic permeability by Evans blue extravasation into PLAT as we previously described [13,14]. Animals were gavaged (1 mL) with Evans blue dye (1%) diluted in water, and 30 min later the animals were sacrificed. PLAT was carefully removed from the mesenterium, freed of connective tissue, large blood vessels, and lymphatic vessels and weighed. PLAT was homogenized in formamide and incubated at 37 °C for 24 h. The Evans blue concentration in PLAT; as an index of MLV permeability, was measured using spectrophotometry at 620 nm and normalized to PLAT weight. An Evans blue standard curve was generated from a 1 mg/mL stock (10, 1, 0.1, 0.01, and 0.001 µg/mL).

### 4.4. Flow Cytometric Analysis

For mononuclear cell phenotyping, 1×10^6^ cells in 100 µL were treated with a cocktail of the following reagents: Live/Dead Fixable Aqua Dead Cell Stain Kit (reconstituted in DMSO) (Thermo Fisher, Waltham, MA, USA) PE-anti-rat CD45 (clone OX1, eBiosciences, San Diego, CA, USA), FITC-anti-rat CD3 (clone IF4, Biolegend, San Diego, CA, USA), APC/Cy7-anti-rat CD4 (cloneW3/25, Biolegend, San Diego, CA, USA), PerCP-anti-rat CD8 (Biolegend, San Diego, CA, USA), BV711-anti-rat integrin αE2 (BD Biosciences, San Jose, CA, USA), and PE/Cy5-anti-rat CD11b (Abcam, Cambridge, MA, USA) and incubated at 4 °C in the dark for 30 min. Following incubation, cells were washed in 2 mL of PBS, centrifuged at 500 *× g* for 5 min, and decanted. Using the FOXP3/Transcription Factor Staining Buffer Set (Thermo Fisher, Waltham, MA, USA), cells were fixed and permeabilized by the addition of 1 mL of fixation/permeabilization working solution (eBiosciences, San Diego, CA, USA), briefly vortexed then incubated for 60 min at 4 °C in the dark. Samples were then washed two times with 2 mL of permeabilization buffer followed by 800 *× g* for 5 min at 4 °C and decanted. AF647-anti-rat FOXP3 (clone 150D, Biolegend, San Diego, CA, USA) was added to the residual volume. Samples were incubated overnight (16 h) at 4 °C in the dark, then washed two times with 2 mL of permeabilization buffer followed by 800 *× g* for 5 min at 4 °C. Pellets were re-suspended in 0.25 mL of PBS for FACS analysis on a BD LSRII flow cytometer using FACSDiva Ver. 8.0.1 software for analysis (BD Biosciences, San Jose, CA, USA).

### 4.5. Glucose Uptake Assay

We used insulin-stimulated [3H]-2-deoxyglucose (2-DG) uptake assay in PLAT explants collected from alcohol- and pair-fed animals [23]. Fat explants were incubated at 37 °C, 5% CO_2_ for 40 min in Krebs-Ringer-Hepes (KRH) buffer (40 mM NaCl, 20 mM HEPES-Na, 2.5 mM MgSO_4_, 1 mM CaCl_2_, and 5 mM KCl) with 0.2% BSA. After two KRH buffer washes, the fat explants were split in half, weighed, and incubated in KRH buffer with or without 100 nM of insulin for 20 min. Fat explants were placed in a cocktail of 9.49 mL of KRH buffer, 0.5 mL of cold 2-DG (2 mM of 2-deoxyglucose in KRH buffer), and 10 µL of radioactive 2-DG ([3H]-2-deoxyglucose) for 3 min at room temperature. Fat explants were washed twice in unlabeled 2-DG, homogenized, and incubated in 0.2 N NaOH for 1 h at room temperature. Scintillation fluid (3 mL) was mixed in 300 µL of the homogenate for 2-DG reading. Radioactivity by scintillation counting was assessed in each sample. Samples were normalized by fat explant weight.

### 4.6. Statistical Data Analysis

Summarized data are indicated in the respective figure legend and are presented as means and standard error of the mean (SEM), the N indicates number of animals. Unpaired T-test was used to detect significant differences between pair-fed and alcohol-treated groups. GraphPad Prism 5.0 software (GraphPad Software, San Diego, CA, USA) was used, and data were considered statistically significant at *p* < 0.05.

## Abbreviations

DCs	Dendritic Cells
Tregs	Regulatory T Cells
MLN	Mesenteric Lymph Node
MLVs	Mesenteric Lymphatic Vessels
PLAT	Perilymphatic Adipose Tissue
MFAT	Mesenteric Adipose Tissue
SFAT	Subcutaneous Adipose Tissue
PBLs	Peripheral Blood Lymphocytes
2-DG	[3H]-2-Deoxyglucose
RT	Room Temperature
IR	Insulin Resistance
SEM	Standard Error of the Mean
